# The right to education and brain health: A priority for advocacy

**DOI:** 10.1002/alz.70221

**Published:** 2025-04-28

**Authors:** Timothy Daly

**Affiliations:** ^1^ UMR 1219 Bordeaux Population Health Université de Bordeaux & INSERM Bordeaux France; ^2^ UMR 5164, ImmunoConcept University of Bordeaux & CNRS Bordeaux France; ^3^ Bioethics Program FLACSO Argentina Buenos Aires Argentina

1

In *Alzheimer's & Dementia*, Gonzalez‐Gomez et al. found that Latin Americans in their cohorts had lower educational levels compared to those in the United States, and that these educational disparities had an important impact on brain structure and function across the lifespan.^1^ This adds to a growing evidence base across Latin American countries, including Brazil,^2^ supporting the notion that education protects the brain and promotes healthy brain aging across the lifespan. Low education attainment was recognized by the 2024 Lancet Commission's meta‐analysis on dementia as the most important modifiable risk factor.^3^

Education is recognized by the United Nations as a universal human right.^4^ But in both Latin America (e.g., Argentina) and the United States, austerity policies are currently eroding this right. Given the importance of adequate education for the brain, dismissing the right to free education until adulthood denies citizens their right to brain health,^5^ with the most disadvantaged most likely to suffer. There is a rapidly emerging interest in brain health advocacy in Latin America^6^, ^7^ and across the Global South.^8^ Data from studies like Gonzalez‐Gomez et al.^1^ should be highlighted in the evidence base for brain health advocacy as part of an emerging rights‐based approach to brain health,^5^, ^9^ because the right to education and the right to brain health are mutually reinforcing, and when one is weakened, so is the other.

Ensuring equitable access to education across the Americas and the world is a matter of human rights and public health, and brain health advocates should be vocal in their support of this universal right.

## CONFLICT OF INTEREST STATEMENT

Timothy Daly has no conflicts of interest to declare. Author disclosures are available in the supporting information.

## Supporting information



Supporting Information
